# The paradox of technology in online education during the COVID-19 pandemic: the experiences of safety and security students in a Dutch university

**DOI:** 10.1007/s10734-022-00971-0

**Published:** 2022-12-09

**Authors:** Anna Matczak, Huseyin Akdogan, Dillon Ashmore

**Affiliations:** Safety and Security Management Studies, The Hague University of Applied Sciences, Johanna Westerdijkplein 75, 2521EN The Hague, Netherlands

**Keywords:** Online education, Higher education, Technology, Student satisfaction, COVID-19

## Abstract

The purpose of this paper is to reflect on the experiences of safety and security management students, enrolled in an undergraduate course in the Netherlands, and present quantitative data from an online survey that aimed to explore the factors that have contributed to students’ satisfaction with, and engagement in, online classes during the COVID-19 pandemic. The main findings suggest an interesting paradox of technology, which is worth further exploration in future research. Firstly, students with self perceived higher technological skill levels tend to reject online education more often as they see substantial shortcomings of classes in the way they are administered as compared to the vast available opportunities for real innovation. Secondly, as opposed to democratising education and allowing for custom-made, individualistic education schedules that help less-privileged students, online education can also lead to the displacement of education by income-generating activities altogether. Lastly, as much as technology allowed universities during the COVID-19 pandemic to continue with education, the transition to the environment, which is defined by highly interactive and engaging potential, may in fact be a net contributor to the feelings of social isolation, digital educational inequality and tension around commercialisation in higher education.

## Introduction

Digital transformation has been entering the scene of higher education for decades now, but it is only recently that it has gained such heightened momentum and attention. One of the consequences of the COVID-19 pandemic was the termination of in-person teaching and the unprecedented use of online education as an exclusive teaching platform on a global scale. By 1 April 2020, this rapid transition to online education had, according to UNESCO, affected 1,542,412,000 learners, which constituted 89.4% of all enrolled learners worldwide (Marinoni et al., 2020). This sudden shift was shaped by the pressing need of educational institutions to act swiftly and move classes from a traditional classroom to the virtual environment—often a daunting challenge (see Chan et al., 2022). The purpose of this article is to discuss students’ experiences with online education, within the wider discourse of a crisis response to an epidemiological risk, which occurred on extremely short notice (less than a week) and without preparation or training of students or staff members. Also, the results of a study conducted in a Dutch university will serve as a snapshot to engage with the general literature on online education and recent trends in higher education.

The purpose of this paper is to reflect on the experiences of safety and security management students, enrolled in an undergraduate course in the Netherlands, and present quantitative data from an online survey that aimed to explore the factors which have contributed to students’ satisfaction with, and engagement in, online classes during the COVID-19 pandemic. Moreover, the authors will also expand upon several non-intuitive findings of this investigation; among them are lower satisfaction with online education among tech-savvy students, greater satisfaction with online education among female students or the challenging task of defining engagement in online education.

The safety and security management programme is built upon three overlapping domains, namely (1) public safety, (2) industrial safety and (3) international security. All domains are taught from an interdisciplinary angle. While the educational philosophy of the university promotes the spirit of applied sciences, which translates into the curriculum being composed of more traditional lectures and practice-oriented group-based projects delivered in collaboration with partners from the professional network, there is no particular teaching methodology that the faculty has been following since the inception of the programme. Prior to the outbreak of the COVID-19 pandemic, no particular online components were used in day-to-day teaching. It was only in direct response to this epidemiological emergency that education in the programme took the form of synchronous online learning with some active elements (e.g. polls, quizzes, simulations, breakout sessions), provided predominantly via a well-known virtual classroom platform. This study was the first educational research project conducted within the programme and does not expand on any previous scholarly work in this setting.

This article adds to the scholarship in two ways. Firstly, it contributes to the existing literature on online education by identifying or confirming variables that influence students’ satisfaction with, and perception of, online education in general. Secondly, it sheds light on the use of technology in education as a response to a public health crisis and explores an underlying paradox through the experiences of safety and security management students in a Dutch university. The first part of this paper provides a review of the literature that informed the research design and the selection of variables tested in the study. The second part explains the research design and methodology. Subsequently, the quantitative findings of the empirical analysis are presented and discussed in the context of the existing literature. Lastly, the paper concludes with a number of reflections and lessons learned from the time of the pandemic as well as implications for future research.

## Literature review

The nature of online education during the COVID-19 pandemic needs to be first contextualised within the broader, global trends in higher education. Although higher education is a vastly segmented sector, which varies from country to country (de Wit & Altbach, 2022:220), the emergence of “mass or massification of higher education” is a result of a number of factors, such as the rapid expansion of student enrolments in universities and colleges, the need to accommodate students who cannot attend campus-based education, the perception of education as a means of social mobility, greater access for more students to university education and the call for education to meet the demands of increasingly complex economies (Altbach, 2015:4). While responding to broader significant changes in societies, the structures and social relations within universities have been transformed by the increasingly prevalent logic of the market and commercialisation, which shifted the narrative about the role of higher education (see Komljenovic & Robertson, 2016; Williams, 2016; Cunha et al., 2020; Zajda & Rust, 2020). While the advance of market-driven policies and practices in higher education has brought an increasing focus on global competitiveness, accountability and efficiency, it has also exacerbated an inherent tension in postsecondary institutions, posing the dilemma of higher education as either a public good or a private commodity (see Scott, 2021).

Another important feature of the changing landscape of higher education is the rapid development of technology and its role in thinking about teaching and learning (Adedoyin & Soykan, 2020; Picciano, 2019). Although online education overlaps with the broader category of distance education, which encompasses earlier technologies such as educational radio, television, videocassette-based courses, with its oldest form being correspondence study, it should be seen as a distinct entity that has provided new models of education (Picciano, 2019). It was the establishment of open universities,^1^ especially the pioneering role and impact of the UK Open University, which fielded innovative solutions and deployed diverse media tools (Cunha et al., 2020; Guri-Rosenblit, 2019). Open universities have innovated through the use of technologies, first with the means of radio television and subsequently with information and communication technologies (ICTs) (Cunha et al., 2020). The development of distance education has undergone many iterations (see Picciano, 2019; Cunha et al., 2020), and has also inspired innovation at/in campus-based universities (Guri-Rosenblit, 2019). Online education has been increasingly seen as an area of significant opportunity for tertiary educational institutions to deal with increasing enrolments and/or government subsidy cuts, to meet the needs of growing and changing student populations (Picciano, 2019).

However, online education has simultaneously gained popularity among some faculty who see this development as a specific mode of education that requires a shift from the traditional roles of “teachers and disciplinarians” to the roles of “facilitators and mentors”, who adopt a non-directive position in leaving students to pace their own learning activities (Wieser & Seeler, 2018). In practice, pedagogically driven online education means a synchronous or asynchronous delivery of classes, guided by a subset of learning theories, offered as stand-alone courses or in combination with in-person teaching and accompanied by online interactive multimedia, internet-based access to resources and computer-mediated communication (Picciano, 2019). Although one of the main advantages of distance education is its ability to accommodate the needs of different types of learners (e.g. women juggling family life and education, prisoners), the most difficult challenges in this domain remain quality assurance, higher drop-out rates and feeling of social isolation (Wieser & Seeler, 2018).

Successful and satisfactory online education is, rather unsurprisingly, achieved through a high level of lecturer involvement and increased effort to make the online curriculum captivating. Studies indicate that well-designed course content, clear instructions, guided discussion, relevant tasks and case studies, timely summative and individualised feedback and facilitated peer interactions are the best measures to make online education a successful experience for students (Chan et al., 2022). To this end, one of the important aspects of online education is, for example, prompt feedback as it prevents student isolation and detachment (Wieser & Seeler, 2018).

Several factors contribute to the perception of online education through a less optimistic lens. These include technology gaps, digital inequalities, limited digital literacy, poor infrastructure, poor institutional planning, poor/limited support for academic staff, insufficient time for course preparation and poor software tools, as well as feelings of isolation (Joksimovic et al., 2015; Picciano, 2019). Furthermore, interactive and engaging content is a matter of interpretation, as lecturers and students might have different views of what constitutes interactivity and engaging class content (Joksimovic et al., 2015). Moreover, to some scholars, the increased educational coverage provided by online education represents a threat to the quality of education and/or incites academics’ fear of loss of control over teaching (Picciano, 2019; Singh & Hardaker, 2014), which might amplify the perception of higher education as a market commodity discussed earlier.

While technological changes to learning environments have led us to rethink pedagogical approaches, the role of the lecturer and the role of the student, it is also worth emphasising that prior to online education, pedagogy was already moving away from the traditional one-dimensional approach of knowledge transmission towards the general idea of teaching as a dialogue and increased student control over individual learning pathways (see Laurillard, 2012; King, 2022).

## The COVID-19 pandemic: online education as an emergency response

A body of literature on the impact of COVID-19 on student performance and experiences is gradually emerging. While the availability of technology has made distance education convenient and accessible during the pandemic, there are a number of challenges for online education that are repeatedly highlighted in the available literature.

Due to the fact that online education was not the default learning environment for the majority of higher education institutions, the rapid response to the COVID-19 pandemic has been described as a forced digitalisation of teaching and learning during (Flores et al., 2021; Jandrić et al., 2020) or emergency remote teaching (Hodges et al., 2020). Distance education requires more time for students and teachers than face-to-face instruction, and the emergency online teaching induced by the COVID-19 pandemic has often been improvised, without guaranteed or appropriate infrastructural and financial support (de Wit & Altbach, 2022). Given this lack of infrastructure, much of the early advice and support for non-expert online teachers focused on the technological tools available in each institution and whether they were considered adequate to support the transition (Rapanta et al., 2020). In a UK study, which examined the experiences of the University of College London university staff during COVID-19, lecturers with prior experience with online education found it easier to adjust to the technological turmoil during the pandemic (Littlejohn et al., 2021). A cursory review of the literature on the use of virtual synchronous online platforms suggests that they can be perceived by lecturers as a helpful facilitative tool. However, their effectiveness hinges upon technological parameters, such as internet access, local network connectivity and bandwidth. In online education, just as in face-to-face, campus-based education, the quality of education, as well as students’ satisfaction and engagement, still strongly depends on the amount of thought that is invested in planning lectures, individual teaching styles and lecturers’ preparedness to transfer knowledge rather than content (Chen et al., 2019; Mendoza et al., 2021).

The available studies of online teaching and learning suggest that lack of training, poor digital literacy, poor internet connections, academic dishonesty and less frequent student–teacher interactions have been problematic (Moralista & Oducado, 2020). A study from Singapore, which evaluated an undergraduate chemistry course during the pandemic, confirms that a more successful online experience was achieved because there was already an active and engaging course design in place before the pandemic and the transition from in-person to emergency remote teaching was guided by a specific conceptual approach^2^ (see Tan et al., 2020). Another study has confirmed that not only institutional and pedagogical responses but also individual self-regulatory and socio-emotional competencies of the students are factors that lead to more positive or negative student experiences of online teaching in times of COVID-19 (Flores et al., 2021). The rapid adaptation to online education has proceeded without considering the essential differences between in-person and online teaching and learning and the different pedagogical frameworks necessary for online education. While online education, in theory, provides broad opportunities for engagement, it is the nature and quality of the engagement and interactions that is often criticised in relation to how education has been delivered during the COVID-19 pandemic (Chan et al., 2022).

Neither students nor lecturers were mentally prepared for such a shift during the pandemic, and both had to adapt to new ways of teaching and learning and to deal not only with the logistical complications but also the COVID-related emotional stress and anxiety. The emerging research shows that university students are one of the social groups at a heightened risk of psychological distress during an emergency situation, and there is evidence of high levels of anxiety and depression among students who were concerned with worsening personal, academic and financial circumstances (Chen & Lucock, 2022; Villani et al., 2021).

The COVID-19 pandemic has affected every aspect of higher education, not only the teaching and learning aspect, but also how higher education institutions are managed (see Abdrasheva et al. 2022). Online education is associated with accessibility, affordability, flexibility, reduced costs and the alleviation of overcrowded classrooms. Hence, it can serve as a convenient panacea in a time of crisis (Dhawan, 2020). To university management, online education represents an opportunity to seize the education market, expand their reach and maximise their profit, which was quickly noticed and defined during the COVID-19 pandemic as a potential opportunity for entrepreneurship education (Liguori & Winkler, 2020; Chan et al., 2022).^3^

It is still too early to assess if—or to which extent—the outbreak of the COVID-19 pandemic has changed the landscape of higher education permanently. The future will tell whether it has created an opportunity to view online teaching and learning as a challenging, but still promising and effective, opportunity, or whether it has introduced irreversible pathologies. The last 2 years has given many academics pauses not only to discuss the nature and consequences of online education but also to reflect on the recent trends, developments and future prospects in higher education as a whole.

## Methods, sample and data collection

Drawing from the literature on online education before and during the COVID-19 pandemic, this mixed-method study had four purposes: (1) to explore students’ experiences of online education, (2) to explore lecturers’ experiences of online education, (3) to explore the variables that affect students’ satisfaction with online education and (4) to explore the variables that affect students’ engagement during online classes. The qualitative perspective of this study, which addressed the first two research objectives, was supported by two exploratory focus groups, one with students^4^ and one with lecturers,^5^ and aimed to inform the survey design. The quantitative perspective, gathered through the administration of a cross-sectional online survey, aimed at addressing points (3) and (4) to understand the correlation between student satisfaction and the level of engagement during online classes in connection with demographic characteristics and learning environments.^6^ A cross-sectional survey was selected as it provides the opportunity to evaluate multiple variables simultaneously.

In the survey, students were asked about their overall programme-wide experience and satisfaction with online education since the beginning of the COVID-19 pandemic.^7^ All lectures and project workshops were moved to the online learning platform Blackboard and delivered in synchronous classes (with about half of the sessions being recorded and made available at a later stage). Attendance in project workshops was compulsory. The first 6 months of teaching online were especially experimental in terms of implementing creative and engaging tools in online classes (e.g. online quizzes, simulations, breakout sessions). Students were predominantly taught the same way (by using the same virtual classroom platform for all courses, live lectures and similar interactive tools). One notably divisive issue among staff was the question of recording lectures. Some teachers decided against it due to privacy, legal and didactic reasons,^8^ while some allowed it in order to alleviate student stress during the pandemic.

The population of interest was composed of undergraduate students from a Safety and Security Management Studies (hereafter SSMS) programme located at The Hague University of Applied Sciences (THUAS), in the Netherlands. The Hague University of Applied Sciences provides education for 26,000 students from more than 140 countries. At the time of the survey administration, there were 455 students enrolled in the SSMS programme. The survey was administered between 9 and 30 October 2020 by sending a direct survey link to students’ university email accounts. There was compensation for some participants in the form of a lottery. Two follow-up emails were sent, and of 455 students, 151 responded to the survey, translating into a response rate of 33%. The sample size was sufficient for a population of 455, which gives a 95% confidence level, and a 6.5% margin of error (Qualtrics, 2020). After the survey was closed, its variables and data were re-coded and uploaded to SPSS. SPSS version 24 was used to conduct all of the quantitative statistical analyses. The analyses consisted of the following steps: (1) computing the descriptive statistics and (2) conducting the multivariate analysis (i.e. multiple regression test). This statistical platform enabled the data to be comprehensively analysed and interpreted in order to establish any correlations between the variables.

The objective of this study was also to inform the SSMS teaching practice and contribute to the discussion about the nature and consequences of online education as a response to a public health crisis. In order to enhance the transparency and validity of the research design, the research team included two SSMS lecturers and one SSMS student.^9^

### Variables and measures

The survey items that were used to measure the variables were constructed purposively for this survey and are shown in Table 8 in the Appendix. The two main dependent variables in the study were “satisfaction with online classes during the COVID-19 pandemic” and “level of engagement during online classes during the COVID-19 pandemic”. Both were latent variables measured with one item using a 5-point Likert scale (from 1 = strongly dissatisfied to 5 = strongly satisfied).

**Fig. 1:**
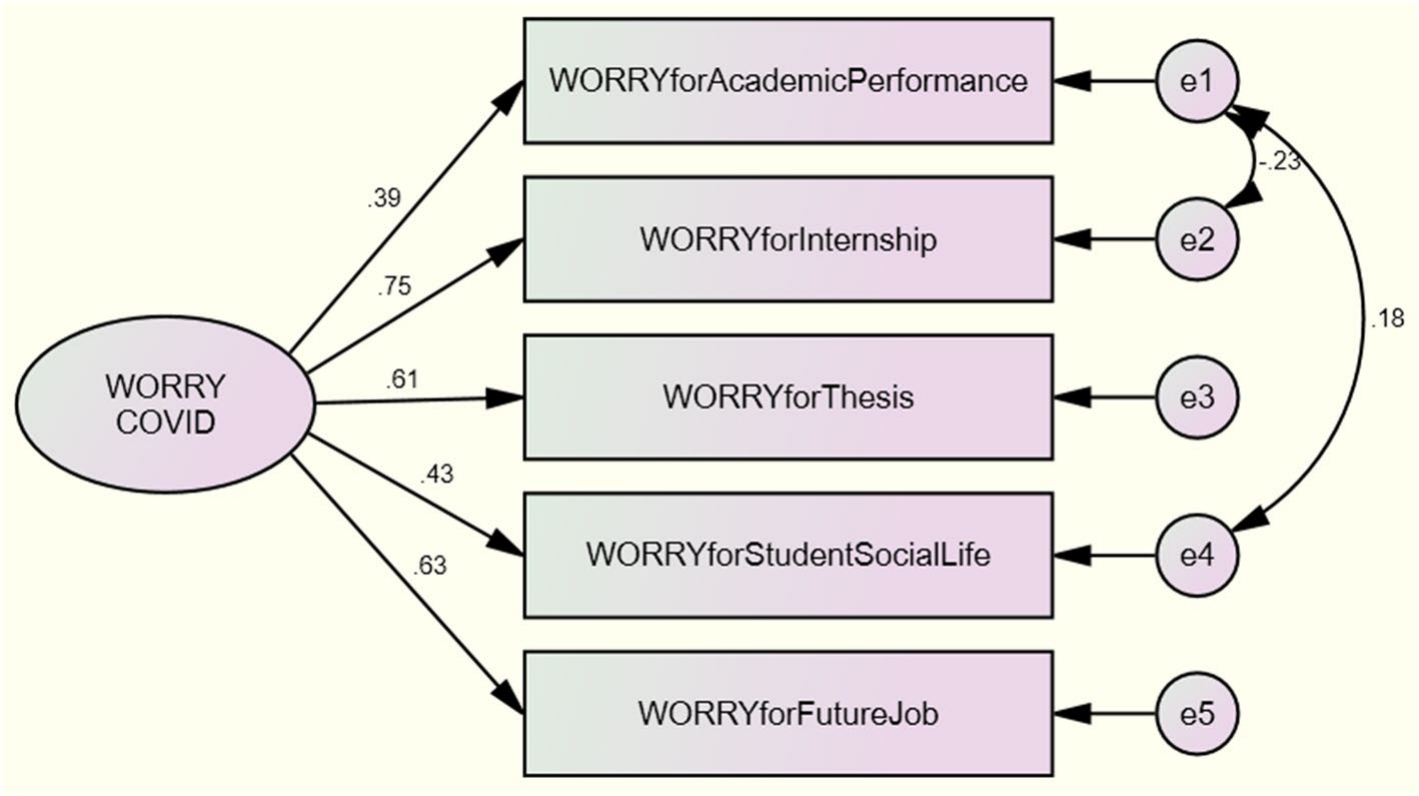
Confirmatory factor analysis (CFA) for WORRY

Among the independent variables were student demographics and different elements of the learning environments prior to and during the pandemic. In order to reflect on the broader context in which online education was pursued, the survey also examined whether students were concerned about their academic performance, thesis completion, internship placement, employability and/or social life. All of the original items were constructed with a 5-point Likert scale (from 1 = strongly disagree to 5 = strongly agree) and, during the analysis, were combined as a latent variable. The validation process to ensure that these items hang together was carried out as follows: to examine the hypothesised structure of this latent variable, both exploratory factor analysis (EFA) and confirmatory factor analysis (CFA) were executed. The results of the Kaiser–Meyer–Olkin (KMO) showed that the sample size was enough for the further analysis (KMO = 0.72). The results of Bartlett’s test of sphericity (*χ*^2^(10) = 126,622, *p* < 0.001) showed that the correlation among the factors was suitable for principal component analysis. An analysis was first performed to obtain the eigen values for each component in the dataset. According to the Kaiser criterion (1), 5 components gave a value above this criterion. These components explain 45.47% of the variance. Confirmatory factor analysis was conducted to validate the measurement model of this latent construct using the AMOS 18 statistical software. Figure 1 shows the CFA model for the WORRY latent variable.

The goodness-of-fit statistics for this model are documented in Table 1, which shows the good fit for this CFA model for the WORRY variable.

**Table 1:** Goodness-of-fit statistics for CFA model for WORRY

Index	Criterion	Model
Chi-square (*x*^2^)	Low	3.633
Degrees of freedom (df)	≥ 0.0	3
Probability	≥ 0.05	0.304
Likelihood ratio (*x*^2^/df)	< 3	1.211
Comparative fit index (CFI)	> 0.90	0.995
Tucker-Lewis index (TLI)	> 0.90	0.982
Normed fit index (NFI)	> 0.90	0.972
Root mean square error of approximation (RMSEA)	≤ 0.05	0.037
Probability (*p* or *p*-close)	≥ 0.05	0.459
Hoelter’s critical N (CN)	> 200	469

The factor loadings were examined to determine strong and weak correlations between the latent variable and its indicators. All of the factor loadings depicted in Table 2 are above the Malthouse’s 0.3 cutoff value. All of the regression coefficients are statistically significant at *p* ≤ 0.001 level. Parameter estimates are provided in Table 2. After the confirmatory factor analysis, the newly structured WORRY variable was ready for reliability analysis. The results of the reliability analysis (Cronbach’s alpha, *α* = 0.684) showed that it is reliable.

**Table 2:** Parameter estimates for CFA model for WORRY

			**UFL**	**SFL**	**S.E**	**C.R**	***p***
WORRY for academic performance	≤	WORRY	1	0.39			
WORRY for internship	≤	WORRY	1.66	0.753	0.49	3.387	***
WORRY for thesis	≤	WORRY	1.258	0.609	0.382	3.293	***
WORRY for student social life	≤	WORRY	1.216	0.426	0.369	3.292	***
WORRY for future job	≤	WORRY	1.584	0.631	0.478	3.31	***

***correlation significant at *p* ≤ 0.001

*UFL*, unstandardised factor loading; *SFL*, standardised factor loading; *SE*, standard error; *CR*, critical ratio

## Findings

### Qualitative data

Although the purpose of collecting the qualitative data was predominantly to inform the development of a student questionnaire, both focus group discussions were recorded, transcribed and coded. Qualitative analysis aims to structure findings by means of using a thematic approach which is based on identifying, highlighting and describing themes that emerge from the data (Strauss & Corbin, 1998). The coding process was carried out independently by the main researcher and the research assistant. The combined, final version of the coding book can be seen in Table 7 in the Appendix. The analysis identified eight main themes: (1) forced start of online education, (2) satisfaction with online education, (3) study performance, (4) facilitation of online classes, (5) paradox of technology, (6) online education—opportunities, (7) online education—challenges, (8) acceptability and future of online education. All themes resonate with the ongoing discussion about the nature of online education during the COVID-19 pandemic, which should be looked at through the emergency lens (Flores et al., 2021; Hodges et al., 2020; Jandrić et al., 2020). There was a broad acknowledgment among the lecturers that the migration to online environment was a self-taught and hectic process. While technology allowed education to continue, it also proved to be an uncharted territory for interactivity and engagement between teachers and students who were not familiar with distance learning and teaching prior to this:

And, uhm, I also think, another issue that I’ve detected is that there is a huge barrier now for students. So, in the beginning I thought that if the student had a question, they’d turn on the mic and ask the question. But is - I mean this must be terrifying, the idea of turning on your mic and speaking into that you know whatever void (…) there’s always this thought, maybe, maybe nobody is listening yeah. It’s kind of like praying to God and you also don’t know if someone is listening. (Lecturer 5)

Due to the limited number of participants, and limited scope of the input from the focus groups, comparisons between the focus groups are not methodologically valid. However, it is worth noting that while students were mostly worried about the effects of the pandemic on their individual health, wellbeing, social life and performance, the lecturers were concerned about the possibility of “Uberisation of teaching” and how the COVID-induced online education might impact the future of higher education in general:

If this new generation sees education as one extra app to have with Netflix, ‘oh I’m gonna [going to] do a bit of education’, then we’re talking about a very different kind of education coming up, eh. And we- I don’t think I’m prepared for that. Because traditional education is also community-based, eh, so they come to school. Also, to know kids of their age, to make friends for life and this kind of thing. If this is gone and now education is [becomes] something you do when you have time or when you’re not working or you took off your morning and you have everything then we need to think it all from scratch (…). It’s like now [turning into] Uber Teacher – choose your teacher based on their rating, be on hold with a teacher who is with a customer now, find an available teacher. (Lecturer 6)

It is beyond the scope of this paper to elaborate more on the qualitative findings; however, some additional excerpts from the group discussions are presented at the end of the article to interpret the quantitative findings and enrich the discussion.

### Quantitative data—sample characteristics

The main sample characteristics are presented in Table 3. The age of the respondents varied from 17 to 31, and most of them were female (58.3%) and international students (51.7%). Students in their 2nd year of study were among the highest cluster (37.7%) of the sample, while only 5.3% of the respondents were in their final (4th) year. About 39.1% of the respondents were working part time and the overwhelming majority of the students (85.4%) had no prior experience with online classes before the pandemic.

**Table 3:** Sample characteristics

Variable		*N*	Percentage
Gender	Male	62	41.1
	Female	88	58.3
	Other	1	0.7
Student status	Dutch	73	48.3
	International	78	51.7
Year of study	1st year	49	32.5
	2nd year	57	37.7
	3rd year	32	21.2
	4th year	8	5.3
Employment	A student and employed full time	6	4
	A student and employed part time	59	39.1
	A student and not employed	75	49.7
	A student and involved in voluntary work	6	4
	Other	5	3.3
Previous experience with online class	Yes	20	13.2
	No	129	85.4
	Do not remember	2	1.3
Age (range: 17–31)	Mean	S.D	
	21.53	2.989	

### Descriptive statistics

Table 4 shows the results of the descriptive statistics. The overall average academic (grade) performance for the respondents was good (*M* = 3.04, SD = 0.901), and their satisfaction with SSMS as a study choice was above average (*M* = 4.36, SD = 0.912). Students were somewhat satisfied with their IT setup (e.g. Wi-Fi, computer, laptop, web camera, microphone) (*M* = 3.98, SD = 0.905), but their experience with Blackboard Collaborate as the main online learning platform provided by the university during the pandemic was very good (*M* = 3.56, SD = 0.990). The study respondents believed that the SSMS lecturers were very prepared (*M* = 4.13, SD = 0.926) to deliver lectures and workshops online during the pandemic, but they found it difficult to say anything about their attention level during the online classes (*M* = 3.17, SD = 1.21) or about their level of engagement with the lecturer and/or with other students during online classes (*M* = 3.15, SD = 1.204). Students rated their comfort with technology above average (*M* = 3.47, SD = 1.142), but their perception of online classes as a convenient solution that allowed them to continue with their education was below average (*M* = 2.31, SD = 1.179). Students neither agreed nor disagreed that online education had allowed for their assessment to be more practical and they were unsure about the impact of the pandemic on their overall educational performance (*M* = 2.9, SD = 1.088). The levels of students’ concern about their academic performance (*M* = 3.46, SD = 1.142), internship (*M* = 3.87, SD = 0.982), thesis completion (*M* = 3.03, SD = 0.920), social life (*M* = 3.69, SD = 1.271) and future job opportunities (*M* = 3.63, SD = 1.117) were all slightly above average. The overall WORRY level based on the aggregated scale variable was also slightly above average (*M* = 3.53, SD = 0.727), meaning that the SSMS students were concerned about the impact of the COVID-19 pandemic.

**Table 4:** Descriptive statistics

Descriptive statistics	*N*	Mean	SD
Average grade performance	151	3.04	0.901
Satisfaction with study choice	151	4.36	0.912
Satisfaction with IT setup	151	3.98	0.905
Level of preparation of lecturers to deliver lectures and workshops online during the COVID-19 pandemic	151	4.13	0.926
Experience with Blackboard Collaborate as the main online learning platform during the COVID-19 pandemic	151	3.56	0.99
Level of attention during online classes	151	3.17	1.21
Level of engaging with the lecturer and/or other students in discussions during online classes	151	3.15	1.204
Comfort with technology	151	3.47	1.142
Convenience	151	2.31	1.179
Assessments becoming more practical	151	3.09	1.139
COVID-19’s impact on educational performance	151	2.9	1.088
WORRY for academic performance	151	3.46	1.142
WORRY for internship	151	3.87	0.982
WORRY for thesis	151	3.03	0.920
WORRY for student social life	151	3.69	1.271
WORRY for future job	151	3.63	1.117
WORRY because of COVID—scale	151	3.5364	0.727

## Multivariate analysis

### Student satisfaction with online classes during the COVID-19 pandemic

The results of the multivariate analysis^10^ for satisfaction with online classes during the COVID-19 pandemic are provided in Table 5. According to the model summary in Table 5, this regression model accounts for 66% of the variance in satisfaction with online classes. Of the independent variables, the regression model calculated that satisfaction with online classes could be predicted based on students’:

- Gender
- Employment status
- Perception of SSMS lecturers to deliver lectures and workshops online
- Comfort with technology
- Perception of online classes as a convenient solution

**Table 5:** Results of multivariate analysis (OLS regression) for the satisfaction with online classes during the COVID-19 pandemic

Model		*B*	Std. error	*β*	*t*	Sig
1	Constant	6.798	0.693		9.804	0.000
	WORRY	0.134	0.083	0.098	1.615	0.109
	Gender	− 0.266	0.111	**− **0.13	− 2.397	**0.018**
	Student status	0.182	0.112	0.091	1.622	0.107
	Employment	− 0.162	0.080	**− **0.118	− 2.04	**0.043**
	Year of study	− 0.105	0.067	− 0.092	− 1.565	0.120
	Housing	− 0.076	0.047	− 0.089	− 1.605	0.111
	Grade	0.072	0.062	0.064	1.161	0.248
	Previous experience with online class	0.017	0.150	0.006	0.115	0.909
	Satisfaction with study choice	0.009	0.063	0.008	0.145	0.885
	Satisfaction with IT setup	− 0.048	0.063	− 0.043	− 0.769	0.443
	Level of preparation of lecturers	− 0.361	0.066	0.315	− 5.429	**0.000**
	Experience with Blackboard Collaborate	− 0.065	0.062	− 0.062	− 1.043	0.299
	Level of attention	− 0.059	0.056	− 0.07	− 1.052	0.295
	Comfort with technology	− 0.324	0.062	**− **0.365	− 5.257	**0.000**
	Convenience	− 0.202	0.059	**− **0.236	− 3.434	**0.001**
	Assessments becoming more practical	− 0.051	0.051	− 0.058	− 0.985	0.327
	R-squared	0.672				

*N*, 151. *OLS*, ordinary least squares; *b*, unstandardised coefficient; *Beta*, standardised coefficient; *SE*, standard error

*p < 0.05; **p < 0.01; ***p < 0.001 (two-tailed tests).

According to the analysis, it was SSMS female students who were more satisfied with the online education during the COVID-19 pandemic (*β* =  − 0.13, *p* = 0.018). The only positive significant finding in the regression analysis is related to the variable that captures students’ perception of lecturers’ preparations to teach in the online environment (*β* = 0.315, *p* = 0.000). When the students’ assessment of the lecturers’ level of preparation score increases on the standard unit, their satisfaction with online classes also increases by 0.315 standard units. The more prepared lecturers were in the students’ opinion, and the more satisfied students were with online classes.

Some of the results were intuitively puzzling. Having the status of an employed student was negatively correlated with satisfaction with online classes (*β* =  − 0.118, *p* = 0.043), meaning the more involved a student was with his/her employment outside of the university, the less s/he was satisfied with online education. There was a statistically significant (negative) relationship between comfort with technology and satisfaction with online classes, holding all other variables constant. For every standard unit of increase in comfort with technology, satisfaction with online classes decreased by 0.365 standard units (*β* =  − 0.365, *p* = 0.000). The more comfortable with technology a student was, the less s/he was satisfied with the online classes. There was also a significant relationship between students’ perception of online classes as a convenient solution and their satisfaction with online education. For every one standard unit of increase in their perception of online classes as a convenient solution, their satisfaction with online classes decreased by − 0.236 standard units (*β* =  − 0.236, *p* = 0.001). The more appreciative a student was of the convenience of online education, the less satisfied s/he was with online classes.

### Level of engagement during online classes

A multiple linear regression was calculated to determine how several factors affected students’ level of engagement with the lecturer and/or other students in discussions during online classes. Of the independent variables which the regression model calculated, the levels of engagement with the lecturer and other students in discussions during online classes could be predicted based on students’:

- Gender
- Level of attention
- Perception of online education as a convenient solution
- Level of worry about their academic, employability prospects and social life

There was a statistically significant relationship between student gender and level of engagement with the lecturer and other students during online classes, holding all other variables constant, suggesting that female students are more interactive (*β* =  − 0.185, *p* = 0.010). In addition to this, there is a statistically significant relationship between the levels of attention during online classes and the level of engagement with the lecturer and other students during online classes, holding all other variables constant. The level of engagement increased by 0.262 standard units for every standard unit increase in attention levels (*β* = 0.262, *p* = 0.003). For every one standard unit of increase in perception of online education as convenient, engagement with lecturers and students in online classes also increased by 0.359 standard units (*β* = 0.359, *p* = 0.000) (Table 6).

**Table 6:** Results of multivariate analysis (OLS regression) for the level of engagement during online classes during the COVID-19 pandemic

Model		*B*	Std. error	*β*	*t*	Sig
1	Constant	0.313	1.072		0.292	0.771
	WORRY	0.278	0.128	0.172	2.167	**0.032**
	Gender	− 0.449	0.171	** − **0.185	− 2.616	**0.010**
	Student status	0.141	0.174	0.059	0.813	0.418
	Employment	− 0.024	0.123	− 0.015	− 0.194	0.846
	Year of study	− 0.012	0.104	− 0.009	− 0.112	0.911
	Housing	− 0.010	0.073	− 0.010	− 0.132	0.895
	Grade	− 0.005	0.096	− 0.004	− 0.054	0.957
	Previous experience with online class	− 0.280	0.232	− 0.085	− 1.206	0.230
	Satisfaction with study choice	0.014	0.098	0.010	0.142	0.887
	Satisfaction with IT setup	0.191	0.097	0.144	1.965	0.052
	Level of preparation of lecturers	− 0.110	0.103	− 0.081	− 1.071	0.286
	Experience with Blackboard Collaborate	0.086	0.096	0.070	0.902	0.369
	Level of attention	0.260	0.086	0.262	3.017	**0.003**
	Comfort with technology	0.176	0.095	0.167	1.846	0.067
	Convenience	0.365	0.091	0.359	4.007	**0.000**
	Assessments becoming more practical	− 0.084	0.079	− 0.082	− 1.055	0.294

*N*, 151. *OLS*, ordinary least squares; *b*, unstandardised coefficient; *Beta*, standardised coefficient; *SE*, standard error

*p < 0.05; **p < 0.01; ***p < 0.001 (two-tailed tests).

The final significant finding of the regression analysis is the independent variable of “WORRY”, created as a scale in this study (*β* = 0.172, *p* = 0.032), and its significant positive relationship shows that the level of engagement of students increased when the level of worry increased, too.

## Discussion

Students’ satisfaction with online classes during the COVID-19 pandemic needs to be contextualised against the broader picture of emergency remote teaching and learning (Hodges et al., 2020) or crisis response to the pandemic, which should not be understood as a natural phase of the global digital transformation of higher education institutions (Adedoyin & Soykan, 2020). While undoubtedly the COVID-induced delivery of online education has brought different experiences for different students (and lecturers), students’ self-reported satisfaction with online education should be seen more as a reluctant acceptance of or settlement with this mode of course delivery, which was introduced as a convenient and quick-to-implement emergency solution.

The five prominent determinants of student satisfaction with online education in our study are students’ gender, perception of lecturers’ preparations to deliver online classes, their employment status and their comfort with technology, as well as their appreciation of online education as a convenient solution during COVID-19. Higher satisfaction with online education among SSMS female students is consistent with other studies on students’ adaptability to online education during the pandemic (Alves et al., 2020; Bisht et al., 2022) and can also be coupled with the findings about a generally higher level of resilience among female students (Allan et al., 2013). The positive correlation between student satisfaction and lecturers’ level of preparation echoes the literature discussed in the introduction to the article and indicates the importance of technological infrastructure, online course design and prior experience with distance education (Chen et al., 2019; Littlejohn et al., 2021; Mendoza et al., 2021; de Wit & Altbach, 2022). The absence of a thorough and well-thought-out design of online classes in the migration process has often led to the rejection of this mode of course delivery as effective online education (Adedoyin & Soykan, 2020).

The negative correlation between the SSMS students’ employment commitments and lower satisfaction with online classes indicates that students who remain in employment throughout their study programme might find it difficult to balance their time between the two. This could be also relevant for in-person teaching. The excerpt from a focus group with SSMS lecturers shows the complexity of the interplay between the perception of online education as a convenient solution, student requests for lecture recording and the temptation to sacrifice education for employment.

Lecturer 1: […] I guess it [online education] was convenient as well as, like, I didn’t have to go anywhere to do the lecture, I could just sit at home and be in bed but then …

[…]

Student moderator: It’s nice but then suddenly you are week seven in and it’s the same setting, and all your friends who you’ve made over the year are these little boxes on screen, there’s nothing else for you to identify them by. And I’d say, like, for me, because I’m an international student like many others who would have gone home when the virus hit [the Netherlands]. […] But suddenly for me, meetups [with friends] became phone calls, if you could, and you’d see friends maybe if they weren’t taking on more part-time work because suddenly, they had the time but then their classes start clashing but because they’re earning money, they’re happy to make the sacrifice.

Lecturer 2 & Lecturer 3: And that’s when they start asking to record lectures.

[…]

Lecturer 4: That’s why I did it, that’s why I recorded lectures. But not now, now you think so, well there were a couple from your group last year who asked me to record them [lectures] because they were working and that’s when I thought, yeah, okay, I can see that, right. Uhm, but I do struggle with it [recording lectures] now because I’m teaching physically and they still ask me to record it and then I think oh they don’t actually want to come to the university even for that one lecture. So, they’d rather stay at home and watch the recording and that defeats the whole purpose.

The COVID-19 crisis was perceived to be likely to exacerbate the global and national inequalities in higher education (de Wit & Altbach, 2022). The greater availability of higher education to less affluent students, the rising costs of university degrees and the increased expectation to have job experience during postsecondary education, all of these, contribute to the fact that employment is a constant feature of student life (Choi, 2018). Student employment remains one of the most important activities that affects students’ academic performance and decisions while in education (ibid.). If the COVID-induced online education created a convenient opportunity for students to shift towards employment, this resonates with a longer and global trend of universities preparing their graduates to be “market ready” (Abdrasheva et al., 2022:12).

Online education is entirely dependent on technological devices and access to the internet. Information and communication technologies, whether in online or campus-based higher education, have long been observed as expanding exponentially and influencing all dimensions of the higher education enterprise (Picciano, 2019). The negative relationship between students’ comfort with technology and their satisfaction with online classes suggests an interesting paradox of technology. In contradiction to what was assumed at the beginning of this research, students’ perception of their technology competence did not contribute to their heightened satisfaction. The finding might indicate higher technological (course design) expectations on the part of tech-savvy students, perhaps combined with deficiencies in the technological skills of lecturers. This stands in opposition to the assumption that the growth of technological innovation and internet accessibility will increase the motivation for online learning. An alternative explanation is that students’ technological skills are confused with their proficiency with social media. Despite the fact that many students are very competent in using social media, they are used for social purposes and communication. This type of competency may give little indication of more general IT skills (Ghosh, 2022).

In the UNESCO-commissioned study, students expect that their campus experience will increasingly be transformed through technology, which can have a positive impact on inclusion and accessibility (Abdrasheva et al., 2022). The evolution of online education, since its inception, shows that there will not be a single uniform trajectory for this transformation (see Picciano, 2019; Cunha et al., 2020). The expansion of technological revolution should, however, be taken carefully, as along with economic, social and cultural capital, COVID-19 has also exposed how the ability to use technical resources to access and process information has become an index of educational inequality (Ghosh, 2022:210).

The findings of our study allow us to rethink the engagement in online classes and successfully narrow down the primary indicators for predicting student engagement. In the SSMS sample, these were (1) gender, (2) attention span during online classes, (3) anxiety level (“worry”) and (4) perception of online education as a convenient solution.

Although the level of student engagement and interaction during online classes represents one of the central elements to the successful experience of education (Wieser & Seeler, 2018), it has been too often oversimplified as a one-way street that squarely puts the burden on lecturers, as opposed to a more complex interaction that involves multiple parties. Engagement is a complex process that involves a sense of affective connection with academic activities and students’ realisation that they are equipped to face the academic demands. Rethinking the role of a lecturer as a facilitator and the student as an independent learner who proactively seeks to optimise her/his learning experience might well be a defining feature of online learning and teaching. However, lecturers’ involvement, support and facilitation of learning during COVID-19 were among the significant factors which differentiated the students who adjusted well to online teaching from those who did not (Flores et al., 2021).

Our research points to the problem of retaining attention during online classes and its potential impact on students’ engagement, an issue that was repeatedly emphasised during the focus groups with SSMS students and staff. The mode of delivery of online classes in the programme was synchronous and many students expressed their preference for recording the sessions (also as a means to address the problem of limited attention spans). However, the lecturers articulated their concerns in relation to privacy rights and uncertainty about how the studied material could be used outside of the dedicated learning environment. This issue is also discussed by Deflem (2021), who argues that teaching in an age of high technology is intertwined with the so-called cancel culture, in which teachers are at a heightened risk of being misunderstood and the content of their lectures can be misinterpreted and distributed beyond their control in unintended ways.

The sudden disruption in the learning environment during the COVID-19 pandemic impacted not only on students’ experience of learning but also on their social and emotional life. Although technology undoubtedly provides a set of tools to strengthen and enrich academic endeavours, as technology has the ability to connect in new ways, it simultaneously bears the risk of disconnection (Wieser & Seeler, 2018). The positive correlation between the level of anxiety and student engagement could have been the result of the sudden disruption in the learning environment, which was also reported in other studies (Chen & Lucock, 2022; Villani et al., 2021). Education is not only about in-class engagement. Social interaction is as important as academic performance, and meaningful learning often occurs when individuals are engaged in social activities such as group work, discussion and collaboration. A new generation of pedagogies, teaching and learning methods is likely to predominantly focus on students’ individuality, their social networks, which might be the key elements of the personalisation process (Cunha et al., 2020). Online educators need to consciously and consistently introduce and sustain various types and levels of engagement in online education, but, to some extent, this has to be accompanied by conscious opportunities for social interaction and engagement. The findings also suggest that the aspect of student anxieties requires more attention. Attention paid to this concern might have been compromised during the COVID-19 pandemic as a result of a rapid migration process of teaching components to the online environment.

Moreover, contrary to original considerations informing the research design, the home/international student status in SSMS did not appear to be a significant independent variable in the analysis. While international students are certainly not a homogenous group (they are driven by various motivations, such as cultural experience, the prestige of a world class university, livelihood opportunities), the COVID-19 pandemic has contributed to their particular vulnerability by limiting the cultural immersion and networking opportunities that studying abroad and internationalisation brings about (de Wit & Altbach, 2022; Ghosh, 2022; Kanwar & Carr, 2020). The silent nature of this variable might be explained through the comment about the uniqueness of the SSMS programme and the close and supportive relationship between the faculty and the students given by one of the lecturers in a focus group:

I think that one of the things about SSMS and some of the more successful programmes within this institution is [that] the ones which have a strong sense of community. And that is something more, that’s what I think you’re saying, we’re worried about that because the reason that we get more students is of course the quality of the education but it is also the experiences that international students have, the friendships that they make, the interaction with the lecturers etc. [Lecturer 3]

Further research needs to be conducted to explore the relationship between the perception of online classes as a convenient solution and student satisfaction as well as student engagement. While the positive relationship between this independent variable and student engagement suggests that the accessibility, flexibility and interactive tools, which online education brings about, might translate into higher student engagement, it does not necessarily reflect their overall satisfaction with online education.

## Limitations

The objective of this cross-sectional study was to explore the experiences of SSMS students with online education during the COVID-19 pandemic. Although two dependent variables were subjected to a comprehensive examination, the study results are limited to a specific student population and teaching environment. While the input from two focus groups was helpful to design the student survey, the size and scope of the focus groups (especially the student group) were very limited. The low participation of students in the online focus group could have been a consequence of reintroducing the lockdown measures in the Netherlands at the time that the focus group was to meet. Since this study included only students from a particular programme offered by The Hague University of Applied Sciences (as a convenient sample to reach and due to financial and resource constraints), the findings of the study are only partially generalisable and neither represent the entire student population of the university nor the Netherlands. Thus, future research could aim for a larger sample size, including other Dutch universities and universities elsewhere in Europe. While the combined variable to measure students’ concerns about future academic performance and employability did not turn out to be particularly significant in this study, more attention could be given in the future to the implications of prolonged online education for students’ mental health and wellbeing.

## Conclusions and implications for further research

The primary aim of this article is to contribute to the emerging educational scholarship on lessons learned for the post-pandemic teaching and learning environment as many elements of how education has been delivered for the past 2 years are likely to stay. This study confirms that student gender can be a significant predictor when student satisfaction and engagement during online classes are researched. Similarly, the readiness and preparedness of lecturers to deliver interesting, engaging and interactive online classes has a positive impact on student satisfaction with education. However, there are 2 dimensions to the paradox of technology observed in this study, which are worth further exploration in future research. Firstly, students with higher technological skill levels tend to reject online education more often as they see substantial shortcomings of classes in the way they are administered as compared to the vast available opportunities for real innovation. Secondly, this study shows the importance of attention and commitment in online education as they improve the willingness of students to engage and have meaningful interactions during online sessions.

The secondary objective of this article is to reflect on the last 2 years of teaching in the postsecondary education sector against the broader, global changes in higher education. Online teaching, despite being a response to an emergency situation, might further contribute to the process of “marketisation” of higher education. If students are no longer required to attend sessions in person and if they are simultaneously presented with competing employment opportunities, this might lower their commitment to the course, make them feel less content with the new learning environment and prioritise employment even more. More research is recommended to explore the motivations and impact of student job commitments on their academic performance and employability. Lastly, as much as technology allowed universities during the COVID-19 pandemic to continue with education, the transition to the online environment, which is defined by highly interactive and engaging potential, may in fact be a net contributor to the feelings of social isolation. While the level of anxiety did not appear as significant as envisaged at the onset of the research process, it is very conceivable that students who expressed a higher level of worry used the online platform not only for educational purposes, but also to seek social interactions with lecturers and fellow students and alleviate the uncertainties triggered by the COVID-19 pandemic. There is a need not only to look into students’ wellbeing during the COVID-19 pandemic, but equally afterwards, to examine the long-term impact of this emergency situation on students’ academic and social prospects.

The role of technology in higher education is widely acknowledged, but it is possibly also at risk of being overrated. Online education depends on technology, which has the ability to connect in new ways, and its role to continue with education, especially during the Covid-19 pandemic, has been repeatedly emphasised. However, it is important to acknowledge that technology simultaneously bears the risk of disconnection and study delay, and it also contributes to the digital educational inequality and tension around commercialisation in higher education.

## Figures and Tables

**Table 7:**
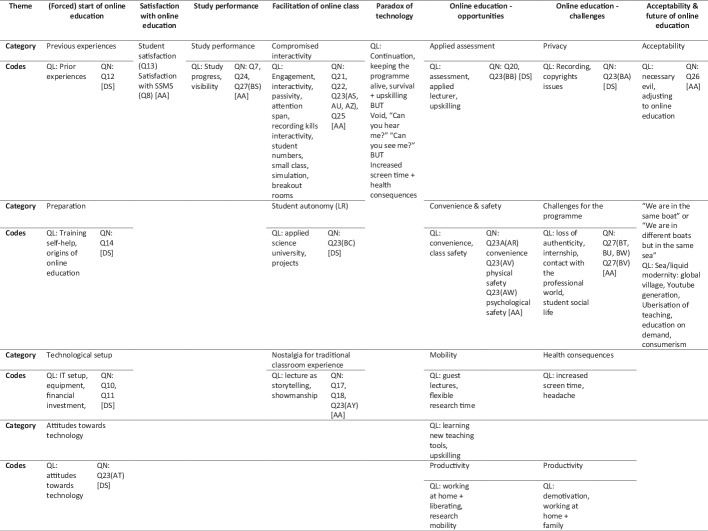
The combined, final version of the coding book

*QL*, qualitative finding(s); *QN*, quantitative finding(s) informed by and/or compared and contrasted with qualitative data; *DS*, descriptive statistics; *AA*, advanced analysis; *LR*, literature review

**Table 8:**
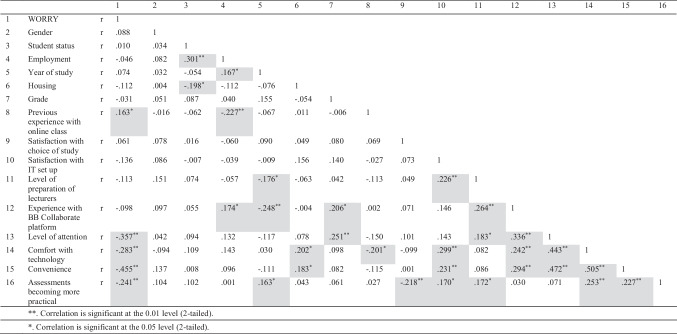
Correlation matrix

## Data Availability

The datasets generated during and/or analysed during the current study are available from the corresponding author on reasonable request.

## Appendix 1

See Table 7

## Appendix 2

See Table 8

## Footnotes

1 In 2018, there were 60 distance teaching universities around the world; among the most well-known ones are the UK Open University, Indira Gandhi Open University in India, Athabasca University in Canada, The Open University of Israel and University of South Africa (Guri-Rosenblit, 2019).

2 During the transition to COVID-induced online education, the coordinators of the evaluated course used the “Community of Inquiry Framework”, which encourages active questioning and enhances student engagement.

3 In the past several decades in many countries, there has been a shift from seeing tertiary education predominantly as a public good to seeing education as a private good, putting more pressure on universities and students to contribute financially to the cost of postsecondary education (Komljenovic and Robertson, 2016).

4 This focus group consisted of three students from three different countries (2 male, 1 female) and was conducted in September 2020.

5 This focus group consisted of seven lecturers (4 male, 3 female) and was conducted in September 2020.

6 Although the study did not receive an official ethics clearance, as at the time of the fieldwork there was no ethics committee launched at the university, the research design and potential risks were discussed with fellow colleagues and the project was carried out according to international research ethics standards (including data confidentiality, informed consent, voluntary participation).

7 The undergraduate programme consists of 32 subject matter courses and 16 group-based projects that at the time of the study were taught by 17 lecturers.

8 The concerns were that (1) students would re-post/publish recordings, (2) legally, lecturers would need to obtain formal consent from all participants for recording, (3) offering recordings would reduce the incentive to join the lecture live and (4) recordings would reduce participation in discussions and make it more challenging for students to contribute.

9 One of the lecturers participated in the staff focus group and was involved in the research process, assisting with statistical analysis of the fieldwork data. The student did not participate in the study and acted as a research assistant throughout.

10 Correlation matrices are shown in Table 8 in the Appendix. The results showed that multicollinearity was not a concern. Assumptions were checked and the tests yielded that there is no violation of assumptions.
